# How do readers choose to undergo treatments based on medical articles?

**DOI:** 10.1097/MD.0000000000005636

**Published:** 2016-12-09

**Authors:** Ye-Seul Lee, Jeongjoo Kim, Seongsu Joo, Byeongho Go, Hyangsook Lee, Younbyoung Chae

**Affiliations:** Acupuncture & Meridian Science Research Center, College of Korean Medicine, Kyung Hee University, Seoul, Republic of Korea.

**Keywords:** cognitive decision-making, health information, medical news articles, pathway analysis, treatment choice

## Abstract

The purpose of this study is to study the reader's cognitive process in reading medical articles and its influence on the decision-making process. Twenty-four participants completed a survey pertaining to 36 medical articles on new treatments with similar text structures and similar titles. Participants rated each article on its level of interest, informativeness, and reliability, and were asked whether they would choose the treatments in the news article. A cognitive decision-making model can be applied to health contexts, in which the reader's subjective ratings on interest, informativeness, and reliability were positively associated with choosing new treatments. The decision-making process path from the perception of informativeness was mediated by the reliability of the news article. Interest was positively linked to informativeness, although it was not directly associated with reliability. This study shows that readers’ subjective ratings on health information can indicate their decision-making. Artifacts in the information that might incite emotions or interest, such as framing, can affect the reader's decision-making process.

## 1 Introduction

Coverage of health-related topics in the news media has increased dramatically in Korea. From the news article database provided by the Korea Press Foundation, it can be determined that the number of news articles, including all newspaper media, containing the term “medical” or “medicine,” was 3,949 in 1990, and increased to 125,524 in 2010, and 221,623 in 2014. This number surpasses a roughly 30-fold increase over 2 decades and shows a dramatic change in information flow regarding medical content available to lay people. Indeed, countless news articles also flow into social media, enhancing the accessibility of health information, including information about new treatments, medicines, and health technologies.^[1]^

Such facts seem to be captured by the expression “the Information Age,” which started in the late 20th century and has continued into the 21st century, and in which the flow of information has been achieved with the help of digitized storage, computers, and the internet.^[2]^ It is now common for people to look to the internet for information if they want to visit a hospital for medical care or search for information regarding a medicine that they are about to take. As the news media have become an important source of information regarding health, medical treatments, and therapies, the intensive and extensive coverage of health issues, including new treatments, has been shown to change the health behaviors of consumers or readers.^[3,4]^ To investigate how health may fall under the social influence of the news media, which represents a primary factor in the adoption and change of health behaviors, recent studies have focused on the quality of reporting.^[5]^ Specifically, previous studies have focused on whether media coverage regarding health information was inaccurate or overly enthusiastic, or whether news articles were framed in such a way as to induce certain emotional effects in the readers.^[6,7]^

Whereas it is widely acknowledged that news coverage of health issues and new medical therapies affects readers, the process of readers’ decision-making upon reading a news article has been only partially studied. Several studies have focused on certain health topics that have generated debate or controversy, such as mammography or influenza A (hemagglutinin type 1 and neuraminidase type 1 [H1N1]), also known as swine flu, by studying whether motivation on the part of consumers had an effect on their attitudes toward news articles and on the knowledge they gained from the news.^[8,9]^ One of the models that has been used as part of this approach is the cognitive mediation model (CMM), or the expanded version of the CMM. The CMM is based on the hypothesis that various motivations lead individuals to pay attention to and to actively process news information, and provides a model for identifying both direct and indirect paths by which individuals acquire knowledge from the media.^[10]^

Despite the fact that an epidemic such as H1N1 or a controversy over medical therapies or diagnostic process such as mammography for breast cancer may motivate the reader to search for more medical news articles, this motivation may not always be the source of behavior change in the current situation in Korea. Considering the large increase in the number of medical articles in recent decades and the easy access to such medical articles over the Internet, it seems that there is already an abundant pool of new information that may lead to health behavior changes in readers. The contents of such news articles may influence the readers’ decision-making process even when readers encounter a series of medical articles by chance. For example, they might become interested at the mention of a new medicine on the front page of an Internet website that they visit every day, even if they have no particular motivation regarding the subject of the news article.

To investigate the possible effect of news articles pertaining to health issues upon readers who have browsed the Internet and read medical articles by chance, this study aims to look at how readers perceive news articles and how these perceptions subsequently influence the readers’ decision-making process with regard to new medical treatments or therapies. Specifically, the research questions were as follows: how do the contents of a medical news article affect the reader's choice-making decisions in choosing new treatments or therapies, and through what process are these factors considered when readers make choice decisions regarding new medical treatment or therapy?

## 2 Methods

### 2.1 Participants

In all, 24 subjects (aged 19–26, average age 22.33, 10 females) participated. Participants were recruited through the Internet, and exclusion criteria were psychological or neurological disorders that might affect their cognitive abilities when reading the medical news articles. Additionally, students with a medical major were excluded because the health literacy of medical students may be different from that of other participants. As the students were healthy nonmedical students, we assumed that the health literacy of the participant group had no significant difference compared with the general population. Before participating in the study, participants received instructions that they would need to complete a survey on 36 medical news articles on paper, all of which pertained to findings on new treatments shown to be effective in a certain disease or disorder. Upon finishing the experiment, the participants received $25. Written informed consent was obtained from participants. This study was conducted in accordance with the guidelines issued by the Human Subjects Committee and approved by the Institutional Review Board of Kyung Hee University in Seoul, Republic of Korea.

### 2.2 Selection of news articles

Articles written between January 2014 and December 2014 that appeared in newspapers in Korea were first chosen as potential candidates. All the news articles were chosen from the website of the Korean Medicine Convergence Research Information Center (www.kmcric.com). To select the final news articles pertaining to medicines, the selection criteria were as follows: all the articles had similar title formats: “*A new drug* proven to be effective in *a disease*”; the quality of the medical news reporting was assessed through a medical news rating instrument, and news articles that did not satisfy at least 6 of 10 standards in the instrument were excluded; the quality of the selected articles showed inter-rater consistency when rated by 2 evaluators (YSL and JK, Cohen kappa value = 0.62). The evaluators selected the final 36 news articles on health issues and medicine, which satisfied all 3 criteria. The order of the articles was then randomized, and subsidiary information such as date or advertisements was deleted so that the participants would only read the title and the content of the news article in the study. The title and the text of all articles were extracted and edited into a uniform format on paper.

### 2.3 Measurements of the cognitive process of the reader involved in decision-making

The survey of this study included 4 questions that asked for the reader's immediate response to each of the news articles upon reading it. At the end of each article, the reader was asked to rate it subjectively along 4 dimensions: whether the news article was interesting; whether it was informative; whether the contents seemed reliable to the reader; and whether the reader would choose to receive the new treatment described if the reader had the disease that was the subject of the news article. All 4 measures were scored on a 5-point Likert-type scale ranging from 1 (strongly disagree) to 5 (strongly agree). These 4 measures became the variables used in the pathway model to explain the cognitive process of the reader in decision-making.

These 4 questions were created in an attempt to measure perceptions of news content as communication, which places importance on the perceived enjoyability of the communication, and does not always require accuracy or truthfulness of its contents. A previous study has suggested that these latter distinctions exist only for writers and researchers, but not for readers.^[11]^ Whereas several previous studies have focused on the quality of the news articles, and many have concentrated on how motivated readers obtain the knowledge they seek in medical news articles, another study hypothesized that since entertainment value and information value are not strictly separated according to entertainment and news coverage, readers’ perceptions and evaluations of news articles may combine elements of both news and entertainment.^[12]^ Indeed, this study showed that in news articles, credibility, emotional liking, quality, and representativeness were the 4 major criteria along which readers perceived and rated news stories.

In addition, there have been critiques of medical news articles being overly enthusiastic regarding new treatments or therapies, which may blur the net effectiveness of the treatment. Such articles focus on inducing interest in readers by using extreme expressions or framing of the contents. News articles have been reportedly framed to induce certain emotional effects in readers.^[6,7]^ Our study was based on a similar hypothesis, which is that readers who are not experts in medicine and who have limited knowledge of such topics may evaluate news articles based on entertainment criteria as much as on informational criteria. Thus, our hypotheses can be set out as follows:

- Hypothesis 1: Reader's interest in the medical news article will be significantly associated with the choice decision.
- Hypothesis 2: Perceived informativeness will be significantly associated with the choice decision.
- Hypothesis 3: Perceived reliability will be significantly associated with the choice decision.

### 2.4 Analytical approach

**Figure 1:**
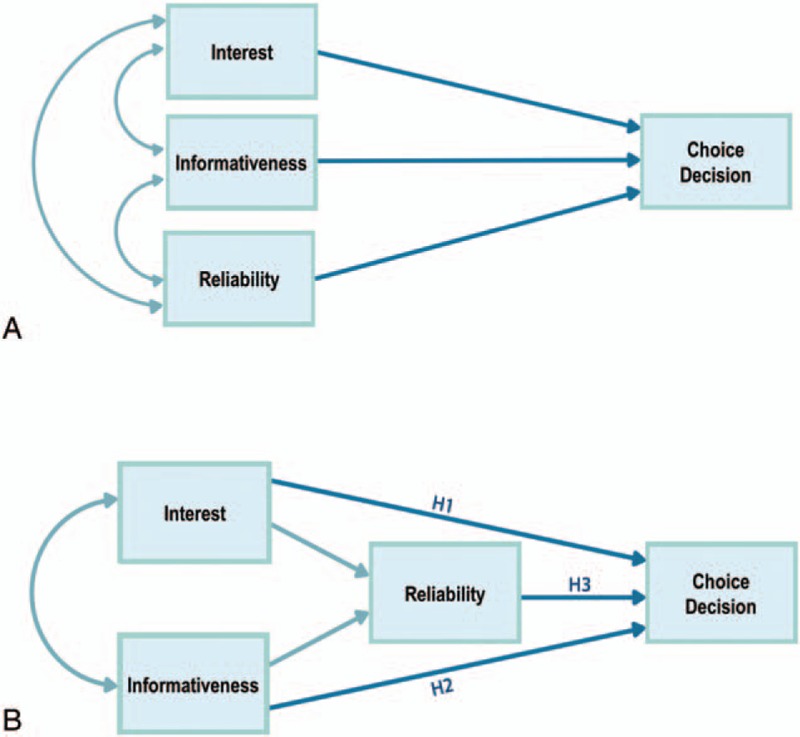
A, Relationship between the variables. Choice decision is the dependent variable, and the other 3 variables are correlated to one another. No specific model to explain the relationship between the variables is visualized. B, Hypothesized model of cognitive decision-making using pathway analysis. Choice decision is the dependent variable, and reliability is associated with interest and informativeness. Reliability is then associated to choice decision. H1 to H3 = hypotheses 1 to 3.

According to our hypotheses, the 4 previously described measures that were obtained in the form of participant ratings are expected to have direct or indirect effects on one another. This relationship is shown in Fig. 1A, where all of the variables are linked to one another with choice decision as the dependent variable. Furthermore, interest level, informativeness, and reliability are expected to be associated with the choice decisions of readers. To test these hypotheses, we used a pathway analysis model to identify the reader's cognitive process in decision-making regarding the new treatments. Pathway analysis is a method that tests a model of direct and indirect effects on an outcome.^[13,14]^ Direct and indirect paths were tested among the variables in our analyses. The saturated (overidentified) model, which includes all possible pathways, is shown in Fig. 1B.

Model fit was analyzed to see whether the proposed model explained the cognitive pathway of the readers when reading the medical news articles. We used 4 fit indices to gauge our model fit: the model chi-square (χ^2^), relative chi-square (χ^2^/df), comparative fit index, and the Tucker–Lewis index. If the model fits the data well, χ^2^ should not be significant. The χ^2^ is divided by its degrees of freedom to adjust the sensitivity to sample size. Relative χ^2^ values that fall between 1.0 and 5.0 are considered a good fit.^[15]^ For the comparative fit index and the Tucker–Lewis index, a value that falls between 0.95 and 1.00 indicates a good fit.^[13]^ For the root mean square error of approximation (RMSEA), a value of 0.05 or below indicates a good fit.^[16]^ The Amos 19.0 software was used for the analysis.

## 3 Results

### 3.1 Variables and model fit

**Figure 2:**
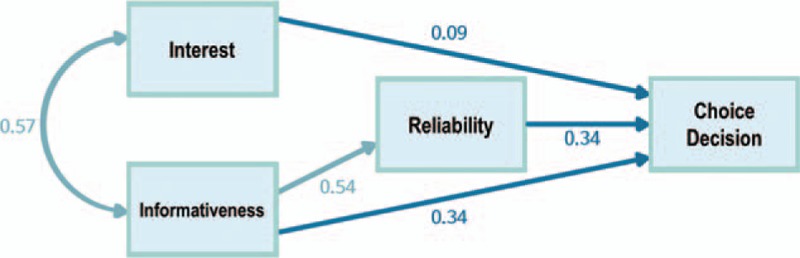
A final model for the cognitive decision-making process using pathway analysis. The coefficients are directional standardized beta coefficients. Significant paths are indicated by solid lines. The variables without solid lines indicate that they do not have any direct relationship between one another after applying pathway analysis.

Bivariate correlations among the variables are shown in Table 1. Looking at the associations among the 4 variables, it can be seen that the 4 variables—interest, informativeness, reliability, and choice decisions—were all significantly correlated with one another. With these 4 variables, we tested our model of cognitive decision-making. Our final pathway model of readers’ cognitive decision-making, which showed choice decision as the consequent variable, was tested for model fit. Our model fitted the data well (*χ*^2^ = 0.86, df = 1, comparative fit index = 0.999, Tucker–Lewis index = 1.000, RMSEA <0.001), meaning that the model explained the cognitive decision-processing of readers upon reading the medical news articles. Figure 2 shows the empirical findings of the model, with the significant paths and their standardized regression weights.

**Table 1:**
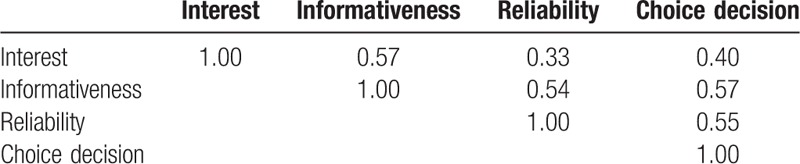
Bivariate correlations among the variables.

### 3.2 Cognitive decision-making process

Hypothesis 1 posited that interest would be associated significantly with the choice decision. Our result showed that the interest level of the medical news article was in fact associated with the choice decision (*β* = 0.094, *P* = 0.003). Hypotheses 2 and 3 posited that through pathway analysis, informativeness and reliability would be significantly associated with choice decision. Both informativeness (*β* = 0.338, *P* < 0.001) and reliability (*β* = 0.336, *P* < 0.001) of the article accounted for the choice in decision-making. That is, if the reader deemed the contents of the medical news article to be informative, then he or she would be more likely to choose the treatment. Similarly, the reliability of the medical news article was positively associated with the choice decision; if the reader considered the medical news article to be reliable, then he or she would be more likely to choose the treatment. These results satisfied hypotheses 2 and 3.

Furthermore, the relationships among the measures associated with choice decision were analyzed. The interest level of the articles, which was measured by asking readers whether they thought the medical news article was interesting to them, was positively associated with informativeness (*β* = 0.543, *P* < 0.001). That is, readers were more likely to find the contents of the medical news article informative if they found it interesting. However, in our model, interest level and reliability had no direct path between them. This indicated that readers’ perception of whether the contents of the news article were reliable had no direct relationship with their perception of the news article in terms of interest or enjoyability, and had only an indirect effect through their perception of the news article's informativeness.

## 4 Discussion

Our study demonstrated that the reader's perceptions of a news article in terms of the reader's interest, perceived informativeness, and reliability of the article were positively associated with the behavioral choice decision. A news article often first interests the reader with the topic and content, inducing him or her to read further, thereby delivering more health-related information to the reader. The decision-making process path from the perception of informativeness was mediated by the reliability of the news article. In this study, the hypothesized model was applicable to the decision-making process in involved in reading medical news articles. Our results show that the model in which the behavioral choice decision was the outcome variable fits the data, and also the saturated model. Furthermore, we demonstrated that reading medical news articles can affect the behavioral choice decision even without explicit motivation.

Processing information from news articles that are framed so as to trigger certain emotions such as anger or sadness has been shown to elicit different effects than news articles without frames; these effects are mediated by increased emotions as a result of reading the article, especially with regard to forming opinions, attitudes, or behavioral intentions toward the topic.^[6,7]^ Health-related news articles that induce emotions such as sadness, fear, or hope may have different effects on the readers’ interest, subsequently affecting their health behavior. Furthermore, studies have shown that news articles on the internet affect decision-making with respect to health issues, communications with doctors, and perceptions of trust regarding the health topic.^[17]^ It has been shown that even the slightest mention of health issues in the mass media can affect a reader's health-related behaviors.^[4]^ Whereas the emotion induced from reading the medical articles may have an influence on the reader's interest, the possible impact of perceived informativeness and reliability on the reader's behavior call for an attention on the delivery of medical information through news articles.

Recent studies have argued that due to changes in the social environment, such as easy access to the internet and social media, people are more prone to being affected by news articles they receive through these channels, leading to changes in their health behaviors. Furthermore, such changes include closer social networks and increased communications, which suggests that their behavior changes are more rapid than before.^[18]^ Past studies have investigated the possibility of the internet as a communication medium for health behavior changes. As the internet features hybrid characteristics of mass media and interpersonal communication, studies have argued that the internet has the potential to be a location for interventions that promote health behavior changes, deliver information, and guide programs for informed decision-making about health-related issues.^[3]^ Indeed, this potential has been realized in recent years, for example, by information pertaining to the H1N1 pandemic, and also by health news coverage related to mammography. Both of these issues involved the internet as a medium of information flow, and also as a search channel for information-seeking readers.^[8,9]^ Additionally, whereas new online articles have demonstrated a third-person effect regarding health information, this effect decreased in high social media metric conditions. That is, when readers were more interested in the health topic, they thought of the health information as something that was highly associated with themselves, which would eventually lead to changes in their own decision-making processes and health behaviors.^[19,20]^

Our study can be applied to different groups of actors involved in medical information delivery, and also medical decision processes. Information providers such as reporters in the news media must be cautious against framing of the medical articles amongst the plethora of information. Whereas medical news articles need interesting formats and titles to receive attention from the readers, they must not be framed to cause bias or incorrect decision from the readers. The readers, on the other hand, must be aware of the information delivered through the context when encountering medical news articles, and make judgments based on the experimental results introduced in the medical articles instead of the aggressive expressions or contexts that was created to influence the readers emotionally. In addition, medical practitioners, and also public health workers must keep an eye on the delivery of medical information through news media. Whereas information delivery through news articles can be immediate and efficient, the content may be subject to frames or inaccurate details. The model in our study can be applied to health contexts in which the reader's subjective ratings of interest, informativeness, and reliability are associated with choosing new treatments.

This study has some limitations. First, the sample size was small, and the participants recruited were within a narrow age range, which may have limited their interest in medicine. Second, because the study used direct questions to collect answers from readers, implicit impressions or responses were not considered. A single-item measure on the 4 domains may lack in reliability and validity in terms of psychometrics. However, this study used instant, explicit perceptions from the readers on a number of medical articles to test our hypothesized model regarding medical news articles and their effects on behavioral choice decisions. The results demonstrated that our model was able to explain the cognitive decision-making process of the readers, and also the saturated model. Additionally, we showed that interest, informativeness, and reliability of the article were important factors that should be considered in evaluating the quality of medical news articles and their possible effects on lay people.

In conclusion, our study shows that readers’ subjective rating of news articles can indicate their decision-making with respect to the new treatment introduced in the news article. The information that the news article provides must be accurate and objective, and not leaning towards sensationalism, and it must be sufficient for a reader who is not a medical professional.^[1,12]^ This study showed that among these factors, perceived informativeness and reliability by the readers were important with regard to choosing the new medical treatment or therapy introduced in a news article. Furthermore, whereas interest and reliability of the news article were not related to one another, there was a significant indirect association, mediated by informativeness, between interest and the behavioral choice decision. Interest level was positively linked to informativeness, although it was not linked to reliability. This shows that any artifacts in the article that might incite emotions or interest, such as framing, can affect the reader's decision-making process on health issues. Both information level and reliability showed direct positive relationships with regard to choosing new treatments.
